# A New Feedback-Based Method for Parameter Adaptation in Image Processing Routines

**DOI:** 10.1371/journal.pone.0165180

**Published:** 2016-10-20

**Authors:** Arif ul Maula Khan, Ralf Mikut, Markus Reischl

**Affiliations:** Institute for Applied Computer Science, Image and Data Analysis Group, Karlsruhe Institute of Technology, Karlsruhe, Baden-Wuerttemberg, Germany; Institute of Automation Chinese Academy of Sciences, CHINA

## Abstract

The parametrization of automatic image processing routines is time-consuming if a lot of image processing parameters are involved. An expert can tune parameters sequentially to get desired results. This may not be productive for applications with difficult image analysis tasks, e.g. when high noise and shading levels in an image are present or images vary in their characteristics due to different acquisition conditions. Parameters are required to be tuned simultaneously. We propose a framework to improve standard image segmentation methods by using feedback-based automatic parameter adaptation. Moreover, we compare algorithms by implementing them in a feedforward fashion and then adapting their parameters. This comparison is proposed to be evaluated by a benchmark data set that contains challenging image distortions in an increasing fashion. This promptly enables us to compare different standard image segmentation algorithms in a feedback vs. feedforward implementation by evaluating their segmentation quality and robustness. We also propose an efficient way of performing automatic image analysis when only abstract ground truth is present. Such a framework evaluates robustness of different image processing pipelines using a graded data set. This is useful for both end-users and experts.

## 1 Introduction

Image processing seeks to find, quantify and classify objects accurately in an image. It is being used in a variety of different application fields such as remote sensing, object detection and classification in manufacturing and data processing. Automated image acquisition systems produce plethora of image data, that for manual inspection become time consuming.

Image processing can be done in a manual or automatic way. Manual image analysis performed by humans delivers reliable results but is time-inefficient and burdensome on big data sets. In this case, automatic image processing algorithms designed by computer programmers can be used to perform an efficient and automated image analysis. Automatic image analysis requires less or almost no intervention from the user, however tuning of parameters can be exhausting.

**Fig 1:**
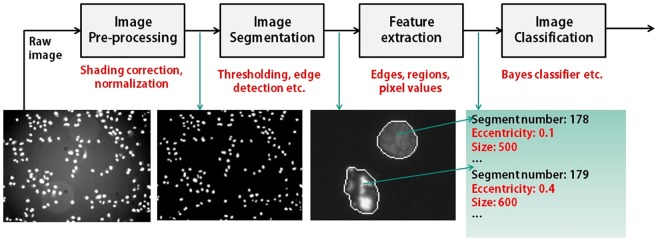
A typical example of image processing pipeline using different image processing steps. The aim is to extract useful objects or regions, features for objects and / or object classification to assign object classes to each segment.

The sum of all processing steps applied to extract the object information from a captured scene is called the image processing pipeline. One typical example of a pipeline for image analysis is shown in Fig 1. Usually, a pipeline consists of the processing steps of pre-processing, segmentation, feature extraction and classification. For each processing step, one or more operators are employed that require suitable parameters.

The pre-processing aims at unifying all images in a sense that noise is suppressed, shading is minimized etc. [1, 2]. The segmentation discriminates between useful objects as foreground and a trivial or unwanted background region. A lot of methods have already been proposed such as thresholding [3], histogram-based segmentations [4], edge detection [5, 6], region growing [7], clustering [8], compression-based, split-and-merge, watershed, graph-partitioning segmentations [9], active contours [10] and many more [11].

The feature extraction quantifies each object i.e. based on shape and intensity [12, 13]. Using a smart set of features, a classifier can trace a set of potentially different targeted objects in an image. Automatic image classification has also been seen in literature. For instance, [14] describes total scene understanding using a hierarchical generative model for 8 different classes depicting sport scenes. In [15], an approach based on supervised maximum likelihood classification was used for change detection to classify different object types from remote sensing data.

Image processing suffers from distortions present in image data sets due to shading, noise, occlusion etc. Artifacts and distortions vary from image to image and make it hard for the user to select global parameters in a feedforward fashion if a large number of parameters is involved. Thus, an operator or a pipeline delivers good results for a specific subset of images. The same parameter set may not work for other images. Therefore, an optimal parameter set for the image processing pipeline is desired.

Parameters for operators are found automatically or set manually based on a subset of an image data set. The manual setting of parameters necessitates knowledge and experience about objects to be found and about the parameters to the algorithm to be used. This a priori information is used to improve parameters in a manual feedback fashion. Thus, not only a single operator such as image segmentation is tuned, also other operators (e.g. classification) of the pipeline can be added. This tuning is done manually and the user evaluates intermediate or final results. Many tools enable and support this process by visualization of results ([16–19]). Evaluation methods for data sets with ground truth are given in [20, 21]. Each selection of parameters gives a certain result that is evaluated according to an evaluation criterion based on data labels / expert inputs. A parameter is said to be optimal according to the problem definition if it yields the best values of evaluation criteria. In the absence of an a priori knowledge, other internal evaluation measures could also be formed.

In the past, automatic methods for feedback-based parameter adaptation in image segmentation have been described in [22–27]. In detail, a genetic approach for adapting the image segmentation parameters using a fitness function for segmentation evaluation in presence of a priori knowledge is given in [28]. More recently, [29] describes automatic principled parameter finding for image segmentation algorithms using visual response surface exploration sampling of parameter search space sparsely and then applying a statistical model for the estimation of the response of a particular segmentation algorithm. In [30], a pulse coupled neural network is used to segment images automatically by adapting the decay speed of a threshold adaptively. [27] uses an offline parameter tuning technique for evolutionary algorithms in the field of image registration.

However, none of the methods allows the insertion of a priori knowledge into the optimization process such as estimated object size and number or distribution of object classes. Furthermore, there is no study evaluating the robustness of optimized parameters depending on noise, artifacts etc.

Previously, we introduced a parameter adaptation technique for image segmentation using a priori knowledge in [31]. It was shown that a standard Otsu segmentation can be improved by adapting its parameters. However, due to the lack of detailed ground truth, the improvement could not be quantified on a bigger scale. To measure segmentation and classification accuracy, a data set must contain both, segmentation and classification ground truth. Recently, we developed a benchmark data set which contains image scenes with ground truth about object position and object class, varying noise and artifact levels [32].

Therefore, the main focus of this work is to introduce a feedback-adaption for the parameters involved in the image segmentation process and discuss the effect on segmentation and classification accuracy with respect to image artifacts and noise. The aim is to show that using feedback parameter adaptation of image segmentation algorithms improves the segmentation/classification outcome in the backdrop of varying artifact levels in comparison to using feedforward algorithms.

This paper is organized as follows: First, image processing goals for two different cases i.e. presence of explicit ground truth and presence of only abstract ground truth, are presented in Section 2. Later, image processing pipeline structures used for these goals are proposed with the evaluation measures and evaluation data set, feature calculation and automatic parameter adaptation of proposed image processing steps are given in Section 2. Results for each case are given in Section 3 followed by conclusions in Section 4.

## 2 Materials and Methods

The scenarios for object segmentation and classification in an image processing pipeline and the aims to be fulfilled in such scenarios are different for a computer programmer. For instance, the success of supervised image segmentation is judged according to the evaluation measures based on a ground truth. However, the ground truth could be absolute in terms of object features and class or it may not be as complete or explicit. Evaluation measures and parameter tuning could potentially be different from each other in such scenarios. These scenarios are divided in cases presented in the following section according to the evaluation of the outcome and parameter tuning schemes.

### 2.1 Case description

The following cases are addressed:

1. In the presence of explicit ground truth, the improvement of standard image segmentation strategies by feedback parameter adaptation has to be investigated. Moreover, segmentation and classification results of image processing algorithms using manually tuned parameters by experts with adaptive image processing parameters need to be compared. The training data set contains a segmentation and classification information for each pixel i.e. 0: background class and 1, …, *K*: object classes (**Case 1**).
2. In the presence of only abstract ground truth defined by end-users, the quality of optimized parameters has to be discussed. Only the approximate number of objects of classes *k* = 1, …, *K* is known and a description which feature ranges for each class are expected (**Case 2**).

**Case 1:** This case deals with automatic feedback-based image processing when explicit ground truth is present. One image processing step, i.e. image segmentation is chosen, and two different standard methods are compared using feedback-based techniques. An image classification criterion is duly integrated into the evaluation criterion for the selection of an optimal parameter set for each individual image. Robustness is given as an average quality value of the outcome over all artifact levels.

One can find a robust parameter set automatically using the feedback mechanism for the whole data set based on the robustness measure that evaluates the quality of images at all artifact levels. One can also tune parameters for each individual image at a varying artifact level and then evaluate robustness of an image segmentation algorithm over the whole data set. The results in both cases (i.e. robust parameter adaptation of the whole data set vs. parameter adaptation for each individual image) are compared based on the robustness values of each segmentation algorithm.

Moreover, parameter tuning using single parameter by an expert is also compared to multiple parameters tuning. This is proposed to show improvements in segmentation quality when multiple parameters are tuned automatically.

**Case 2:** In this case, it is assumed that no explicit ground truth is available which is normally the case in many real applications. So, objects to be found are based on user apriori knowledge. One object class was used for proof of principle and it was defined by abstract features provided by a user.

It is more suitable to use feature-based quality methods in the presence of abstract ground truth. This could be the knowledge about object size, shape and intensity etc. Let us say, that a user would like to find objects that are between 100 and 200 pixels having an intensity between 0.7 and 0.9 (when image is normalized between 0 (darkest pixels) and 1 (brightest pixels)). However, such an abstract ground truth can assume a different confidence value (used to check the appropriateness of the segmented object) for different feature values.

Once the feature vectors and evaluation criteria is designed, standard segmentation methods are again compared to feedback-based methods.

### 2.2 Evaluation data set

A benchmark data set (https://sourceforge.net/projects/gait-cad/files/Benchmarks/hardware_items/) is specifically designed to conform to evaluation criteria that are most suitable for our methodology. A complete description of data set is given in [32]. It is based on 4 scenes *r* = 1, …, 4 containing solid objects i.e. fastener and clips. An aggregation of shading level *b* = 1, …, *B* and artificial background Gaussian noise *n* = 1, …, *N* is used to describe the artifact level *A*(*r*, *b*, *n*) for each individual image. The values of *B* and *N* are 13 and 14 respectively. The benchmark is provided with ground truth in terms of object area and type.

### 2.3 Image processing pipeline

For simplicity, we define an image processing pipeline with three image segmentation operators i.e. convolution, thresholding and opening. Therefore, the parameter vector **p** = [*w* *t* *s*]^*T*^ constitutes of three parameters: *w* defines a symmetric *w* × *w* convolution filter with elements equal to 1/*w*^2^. The thresholding operation is applied using an image intensity threshold *t*. Finally, an opening filter with the disk size of *s* of the structuring element is employed.

**Fig 2:**
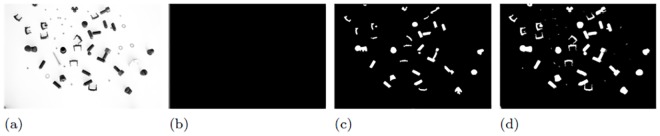
Segmentation results using parameter set p = (*w*, *t*, *s*)^*T*^ (image convolution filter size *w*, intensity threshold *t* and size *s* of a structuring element for image opening). Fig 2(a) is the input grayscale image and Fig 2(b),(c) and (d) show segmentation outcomes using **p** = (1, 20, 1)^*T*^, (3, 120, 5)^*T*^ and (1, 180, 1)^*T*^ respectively.

The effect of a manual parameter tuning by a programmer in case of small artifacts is shown in Fig 2. Obviously, a parameter set of **p** = (1, 180, 1)^*T*^ delivers the best outcome (see Fig 2(d)). However, this may not be the optimal parameter set **p**_opt_.

**Fig 3:**
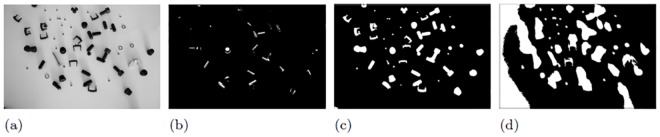
Segmentation results under high artifact levels (i.e. presence of both shading and background noise) using manual selection of p keeping parameters set the same for Fig 3(b),(c) and (d) as in Fig 2(b), 2(c) and 2(d) respectively.

From Fig 3, it is clear that if a high artifact level is present, we may need a different **p** to get a good segmentation result. This is due to the fact that some shading effects are also interpreted as objects. Consequently, different object features and an incorrect object annotation is obtained using the supposed optimal parameter set **p** = (1, 180, 1)^*T*^. A better parameter set could be **p** = (3, 120, 5)^*T*^ at this artifact level as shown in Fig 3(c). In this **p**, *t* was chosen to be lower than 180 since at lower values, the shadows of objects are not detected. Similarly, higher *s* would allow removal of smaller noise BLOBs segmented by using lower *t* value. Here, changing *w* does not impact the segmentation outcome significantly. To find the optimal parameter set is almost impossible for the programmer if the image data set is big or lot of parameters are involved.

### 2.4 Evaluation measures

Measures based on a given ground truth previously used in [32] are segmentation measures (*q*_1_ and *q*_2_) and a classification measure (*q*_3_). Segmentation measures penalize the difference between objects detected and the number of non-overlapping pixels with respect to the ground truth. The classification measure is based on the number of misclassified pixels with respect to the ground truth. These measures (*q*_1_, *q*_2_ and *q*_3_) are converted to fuzzy functions using criteria given in [32] and are represented as *μ*_1_, *μ*_2_ and *μ*_3_. An overall quality criterion *Q*(*r*, *b*, *n*) based on measures given in [32] is:

$$Q\left(r,b,n\right)=\mu_{1}\cdot \mu_{2}\cdot \mu_{3},$$ (1)

and a robustness measure (*R*) according to [32] is given as:

$$R=\frac{\sum_{b=1}^{B}Q\left(r,b,b+1\right)}{B}.$$ (2)

One can also use standard image segmentation evaluation measures like Rand Index (RI), Jaccard Index (JI), Normalized Sum of Distances (NSD) and Hausdorff Metric (HM). However, measures based on true positive and negatives are intentionally avoided as they heavily tend to weight the background which in our case needs not to be segmented. Since a very high number of background pixels are present in the data sets, RI would assume higher values even if no foreground object is detected by the algorithm. Therefore, a criterion Eq (1) described above based on the foreground objects was adopted.

### 2.5 Feature calculation

Targeted features may be geometrical (area, eccentricity, etc.), intensity related (brightness, noise, etc.), and/or content-based (e.g. number of sub-fragments etc.) for each segment type. Feature extraction is of primary importance since object classification is done in the feature space. Each selection of **p** in image processing pipeline yields specific number of segments in each image. These segments may be different in size, extent, underlying pixel values etc. from the segments obtained at another **p** selection.

A computer programmer can also make use of knowledge about the segment features to be found in a data set in an abstract way. The ground truth is described by features (e.g. mean object size, number of objects etc.) desired to be seen in different object types. A criterion based on the feature vector **f**_*i*_ = (*f*_*i*1_, …, *f*_*im*_), considering *j* = 1, …, *m* number of features for each segment *i* where *i* = 1, …, *n*_*t*_ needs to be built. The count of objects found is denoted as *n*_*t*_. The total number of segments *n*_*c*_ to be found in an image can be an additional a priori knowledge.

### 2.6 Automatic parameter adaptation

Two strategies for the automatic parameter tuning are possible: a) One robust parameter set for all images b) Parameter adaptation for each image individually. Parameter adaptation is done differently depending upon the type of ground truth available. When absolute ground truth (for each pixel a segment and class assignment is given) is present, our evaluation measures for BLOBS dependent on **p** can be represented as *μ*_1_(**p**), *μ*_2_(**p**) and *μ*_3_(**p**) in fuzzy terms. Therefore, a total quality measure for each **p** value is given as:

$$Q\left(r,b,n,p\right)=\mu_{1}\left(p\right)\cdot \mu_{2}\left(p\right)\cdot \mu_{3}\left(p\right).$$ (3)

The criterion Eq (3) needs to be maximized with respect to **p** to obtain **p**_opt_(*r*, *b*, *n*) in the case of individual image adaptation and the equation is given as:

$$p_{\text{opt}}\left(r,b,n\right)=\arg\max_{p}Q\left(r,b,n,p\right).$$ (4)

Criterion Eq (1) is used in Eq (4) for parameter adaptation when the deviation of the object feature vector is not under consideration (**Case 1** in Sec. 3).

Besides, quality criteria based on user input as an abstract ground truth can also be formulated using fuzzy functions (requirement in **Case 2** in Sec. 3).

For calculating a quality criterion *Q*_feat_ based on the object feature vector **f**_*i*_, alphanumeric reference features are provided by the user. A user could derive these features based on fuzzy knowledge about the object features to be found. These features are described in terms of fuzzy membership function denoted by *θ*. *θ* is described by trapezoidal fuzzy membership function using Eq (5), where (*a*_*j*_, *b*_*j*_, *c*_*j*_, *d*_*j*_) are edges of a trapezoidal function.

$$\theta \left(f_{ij};a_{j},b_{j},c_{j},d_{j}\right)=\left\{\begin{array}{cc} 0, & f_{ij}<a_{j}\\ \frac{f_{ij}-a_{j}}{b_{j}-a_{j}}, & a_{j}<f_{ij}\leq b_{j}\\ 1, & b_{j}<f_{ij}\leq c_{j}\\ \frac{d_{j}-f_{ij}}{d_{j}-c_{j}}, & c_{j}\leq f_{ij}\leq d_{j}\\ 0, & d_{j}<f_{ij}\;\text{.} \end{array}\right.$$ (5)

The trapezoidal curve given by Eq (5) is a function of *f*_*ij*_, and depends on four scalar parameters *a*_*j*_, *b*_*j*_, *c*_*j*_, and *d*_*j*_. These values are set based on the abstract feature information by the user. For each feature value in the segmented object, a membership value *θ* is evaluated using trapezoidal fuzzy function. For example, if the area is the targeted feature *f*_*ij*_ of the desired object and suitable area values are between 100 (*b*_*j*_) and 200 (*c*_*j*_) pixels, then any segmented object *i* having values between *b*_*j*_ and *c*_*j*_ will get a *θ* value of 1. Then, adding more information by the user is also necessary as in what are unacceptable objects. Consider, that objects below 50 pixels (designated as parameter *a*_*j*_) and above 250 pixels (designated as parameter *d*_*j*_) are worthless. Consequently, *θ* will be 0 outside these boundaries.

The total reference count *n*_*c*_ can also be formulated in fuzzy terms as *θ*_*c*_ according to Eq (5) using (*a*_*j*_, *b*_*j*_, *c*_*j*_, *d*_*j*_) provided by the user as an abstract ground truth. Therefore, according to our measure given in [31], *Q*_feat_ based on fuzzy membership functions in terms of the parameter vector **p**_user_ could be written as:

$$Q_{\text{feat}}\left(p_{\text{user}}\right)=\frac{\theta_{c}\left(p_{\text{user}}\right)}{n_{t}\left(p_{\text{user}}\right)}\sum_{i=1}^{n_{t}\left(p_{\text{user}}\right)}\left(\prod_{j=1}^{m}\theta \left(f_{ij}\left(p_{\text{user}}\right),a_{j},b_{j},c_{j},d_{j}\right)\right),$$ (6)

where, *n*_*t*_(**p**_user_) is the number of objects segmented based on **p**_user_. The summation term in Eq (6) represents the collective deviation of all the object features from the abstract ground truth features. Criterion Eq (6) also needs to be maximized with respect to **p**_user_ as used previously in [31] to get the optimal parameter set **p**_opt,user_ as given below:

$$p_{\text{opt,user}}\left(r,b,n\right)=\arg\max_{p_{\text{user}}}Q_{\text{feat}}\left(p_{\text{user}}\right).$$ (7)

Subscript “user” in criteria Eqs (6) and (7) refers to the optimal parameter set according to abstract user-defined ground truth. Criterion Eq (7) is used for parameter adaptation for each individual image in the presence of abstract ground truth provided by the user. For calculation of a robust parameter for the whole data set, one can use:

$$p_{\text{rob}}=\arg\max_{p}R\left(p\right)$$ (8)

where, **p**_rob_ represents the robust parameter set for the whole data set containing increasing artifact levels.

### 2.7 Feedback adaptation of the processing pipeline

**Fig 4:**
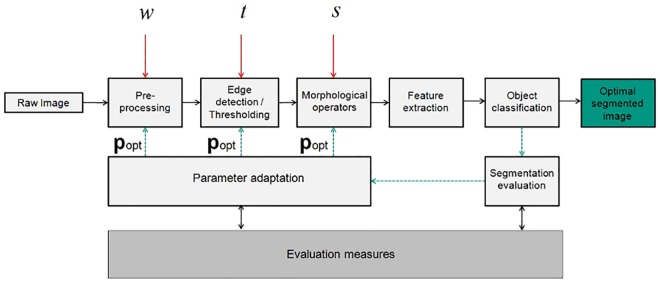
Exemplary feedforward pipeline vs. modified pipeline for the parameter adaptation of segmentation methods for benchmark images. An input grayscale image is first pre-processed to remove noise and shading (parameter *w* is used to affect the pre-processing outcome). The pre-processed image is then used for image segmentation using either edge detection or intensity thresholding (thresholding parameter *t* is used in this step). The segmented image is post-processed using morphological operators to remove too big/too small objects (parameter *s* defines a structuring element for image opening). Features are then extracted from the remaining objects and fed into a classification routine. This pipeline could be modified using structural changes/ parameter adaptation, where evaluation measures are used for segmentation evaluation in order to calculate optimal parameter set **p**_opt_. Using **p**_opt_, an optimal image segmentation is obtained.

An adapted pipeline to achieve automatic feedback parametric tuning is shown in Fig 4. This contains the feedback structure in comparison to the pipeline described earlier in Section 2.3. It shows a feedforward image processing pipeline structure and the new elements for parameter adaptation. Two segmentation methods were adapted and parameters were varied for two different cases. The structure of morphological operators and pre- and post-processing of the images remained identical in both cases (i.e. with and without feedback) when comparing standard segmentation methods. Later, for finding specific objects in an image, only parameter *t* mentioned in Fig 4 was used. Later, all parameters given in Fig 4 were tuned simultaneously to show improvements over single parameter adaptation (Case 1). In the feedback method in reference to expert segmentation, only one parameter was adapted in each method to show the proof of principle (Case 2). The feedback parametric adaptation method for the given pipeline is employed to enable a computer programmer to tune the image processing parameters automatically in cases where the data set becomes large or the number of tuning parameters involved is high enough to be tuned manually. This scheme has been implemented in the Gait-CAD software [33] developed in MATLAB.

## 3 Results

### 3.1 Case 1

Case 1 is useful when a user has a large number of images with an absolute ground truth and a lot of objects are to be segmented in them. The manual parameter tuning would be hectic even with the standard methods. A user can mark a set of images for the available ground truth and adapt parameters automatically using feedback mechanism based on the individual images with varying artifact levels.

This case compares strategies for parameter adaptation described in Section 2.1. Firstly, the whole data set is taken to adopt the robust parameter set that works best at all artifact levels. Then, optimal parameter set is adapted for each individual image using the feedback mechanism. The main aim of Case 1 was to show the performance improvement in image processing (i.e. image segmentation) using feedback-based parameter adaptation with a given absolute ground truth. This is done for the two selected segmentation methods i.e. Otsu segmentation and Sobel edge detection.

Moreover, one can also compare results from an image processing expert doing manual tuning with results emanating from automatic feedback-based tuning. However, as said earlier, manual tuning of several parameters simultaneously is a very tiring and time-consuming task especially in a data set with a lot of information. So, one cannot give a direct comparison for an expert tuning several parameters for each individual image in this data set. Nevertheless, an expert can possibly tune most affecting parameter for each individual image in an image processing pipeline manually. Hereby, we give a comparison between an expert tuning one parameter for segmentation against tuning multiple parameter automatically using feedback in the presence of absolute ground truth.

The input data set used consists of image series defined by *A*(1, *b*, *b* + 1) for all *b*. This ensures stepwise increase in both, noise and shading level at each successive image. In order to adapt one robust parameter set for the whole image series *A*(1, *b*, *b* + 1), criterion Eq (8) was used. However, the parameter set for an image segmentation method was limited to one parameter i.e. threshold *t* such that **p**_rob_ = *t*_otsu_ for Otsu segmentation and **p**_rob_ = *t*_edge_ for Sobel edge detection. *t*_otsu_ is the global gray level threshold for minimizing the intraclass variance between black and white pixels in standard Otsu’s method. This may or may not be an appropriate threshold value for a given image. Therefore, an automatically selected threshold value also denoted as *t*_otsu_ is used in the feedback-based method hereby called as AutoOtsu. *t*_edge_ is used for thresholding the calculated gradient magnitude of the image intensity.

Additionally, a predefined structuring neighborhood window of 3 × 3 was used to dilate the image in case of Sobel edge detection. This was done to ensure that suitable objects are obtained after edge detection. The 5 features selected (*m* = 5) for object classification are: area *f*_*i*1_, eccentricity *f*_*i*2_, solidity *f*_*i*3_, extent *f*_*i*4_ and minor axis length *f*_*i*5_.

**Fig 5:**
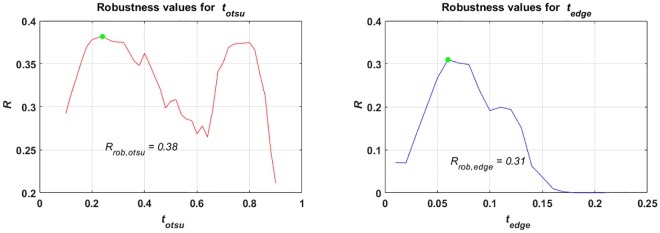
(Case 1) Results for the selection of robust image segmentation parameters i.e. *t*_otsu_ in case of Otsu segmentation and *t*_edge_ in case of Sobel edge detection for whole data set (*r* = 1). *R* vs. *t*_otsu_ for the figure on top and *R* vs. *t*_edge_ for the figure at the bottom. The green dot in both figures represents robust selection of the respective parameters i.e. *t*_rob,otsu_ = 0.24 for Otsu segmentation and *t*_rob,edge_ = 0.06 for Sobel edge detection.

**p**_rob_ was adopted according to Eq (8) for both methods and results are shown in Fig 5. The optimal values of **p**_rob_ are: *t*_rob,otsu_ = 0.24 for Otsu segmentation and *t*_rob,edge_ = 0.06 for Sobel edge detection.

**Fig 6:**
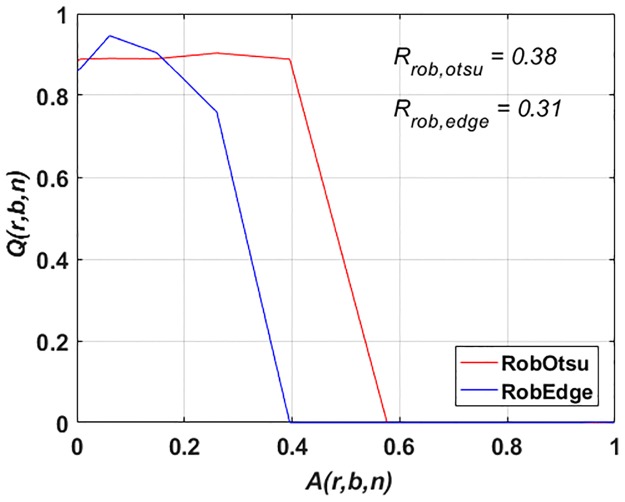
(Case 1) Results for *Q*(*r*, *b*, *n*) vs. *A*(*r*, *b*, *n*) and robustness with the selection of robust image segmentation parameters for Otsu segmentation (RobOtsu) and Sobel edge detection (RobEdge) for the whole data set (*r* = 1). The robustness values are given in Table 1.

Using **p**_rob_, the image segmentation is performed on all artifact levels *A*(1, *b*, *b* + 1) to evaluate and compare the outcome (see Fig 6). Fig 6 shows that at higher artifact levels (*A*(1, *b*, *b* + 1) ≥ 0.3), the robust parameter set for Sobel edge detection produces no meaningful segmentation outcome. On the other hand, Otsu segmentation also does not produce adequate quality at varying artifact levels. At very low artifact level, the quality of the outcome using Otsu segmentation is not the highest in the complete data set. So, to select one robust parameter set for the whole image series with *A*(1, *b*, *b* + 1) is not beneficial at varying artifact levels. Conversely, a computer programmer could look at each image independently to choose a good parameter set according to its artifact level. It could then be argued that one can tune the parameters for each individual image having a distinct artifact level to get better results. An artifact level of an unseen image must be estimated from data to select the appropriate parameter set. Such an estimation could be based on a mean or median value of an image given that the objects to be segmented are in the foreground.

**Table 1:** Robustness values of segmentation methods for different implementations of Case 1 (using explicit ground truth).

Algorithm type	Robustness symbol	Robustness value
RobOtsu	*R*_rob,otsu_	0.38
RobEdge	*R*_rob,edge_	0.31
StdOtsu	*R*_std,otsu_	0.38
StdEdge	*R*_std,edge_	0.44
AutoOtsu	*R*_auto,otsu_	0.77
AutoEdge	*R*_auto,edge_	0.55
MultiAuto	*R*_multi,auto_	0.85
OneUser	*R*_one,user_	0.73

Based on the image processing pipeline given in Fig 4, we then applied the parameter adaptation of both Otsu segmentation and Sobel edge detection to the data set (*r* = 1). For Otsu segmentation, the intensity threshold *t*_otsu_ was used. This parameter was adapted iteratively in order to see the improvement in the segmentation outcome. For Sobel edge detection, also one parameter was used for the standard application and for automatic parameter adaptation.

The standard implementation of both methods are referred to as feedforward application of these methods. StdOtsu and StdEdge are the abbreviations used for feedforward Otsu segmentation and feedforward Sobel edge detection respectively. In standard implementation, parameter values for *t*_otsu_ and *t*_edge_ are not set manually, rather they are being automatically selected by the individual methods. However, these values are not varied in the feedforward method to observe the improvement in the segmentation result.

For parameter adaptation, an exhaustive search was performed using a step size *δ* within the bounds of *t*_otsu_ and *t*_edge_. Since the intensity values of the image dataset is normalized between 0 and 1, the highest and the lowest parameter value should be specified within these bounds. The lower bound used for AutoOtsu was *t*_otsu,low_ = 0.1 and the higher bound used was *t*_otsu,high_ = 0.78 where as *δ* = 0.02. In the case of AutoEdge, *t*_edge, low_ = 0.01 and *t*_edge,high_ = 0.21 where as *δ* = 0.01. *δ* should be *t*_otsu,low_ < *δ* < *t*_otsu,high_ for AutoOtsu and *t*_edge,low_ < *δ* < *t*_edge,high_ for AutoEdge.

In feedback, *t*_otsu_ and *t*_edge_ were adapted iteratively based on Eq (4). Segmentation results were obtained using individual **p**_opt_(*r*, *b*, *n*) for each method at each *A*(1, *b*, *b* + 1). The abbreviations used for feedback-based Otsu segmentation and feedback-based Sobel edge detection are AutoOtsu and AutoEdge respectively.

**Fig 7:**
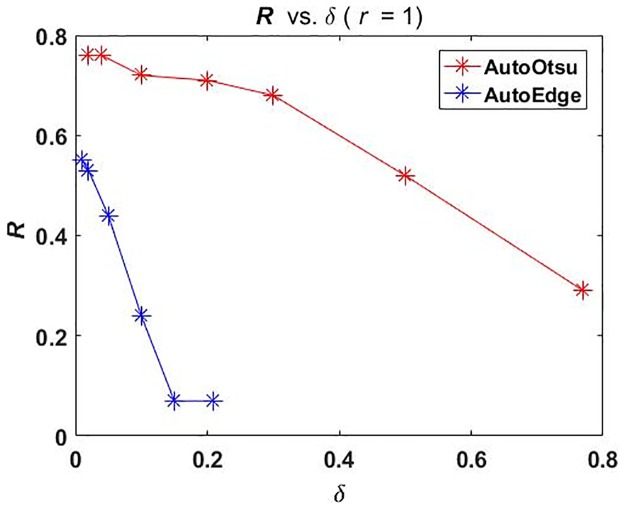
(Case 1) Results of changing *δ* on image segmentation parameter adaptation for benchmark data set *r* = 1. *δ* vs. *R*. *R* values against increasing *δ* for AutoOtsu (red) and for AutoEdge (blue).

The choice of *δ* affects the delivered outcome significantly. Choosing high *δ* will deteriorate the results for exhaustive search and selecting a very low value would make the optimization procedure slow. The aim here is not to show the optimization of adaptation procedure rather the focus is on how to use a feedback technology in order to improve the segmentation results. However, a certain selection of *δ* is necessary to be demonstrated to emphasize on a good adaptation practice. The effect of increasing *δ* on segmentation quality for *r* = 1 is shown in Fig 7.

Fig 7 shows that optimization could be trapped in local optima if the selection of *δ* is not suitable. If *δ* is increased, the quality starts to deteriorate for both methods as indicated by *R* values. However, this effect is much more evident in the case of AutoEdge where *δ* > 0.05 nullifies the use of feedback technology.

**Fig 8:**
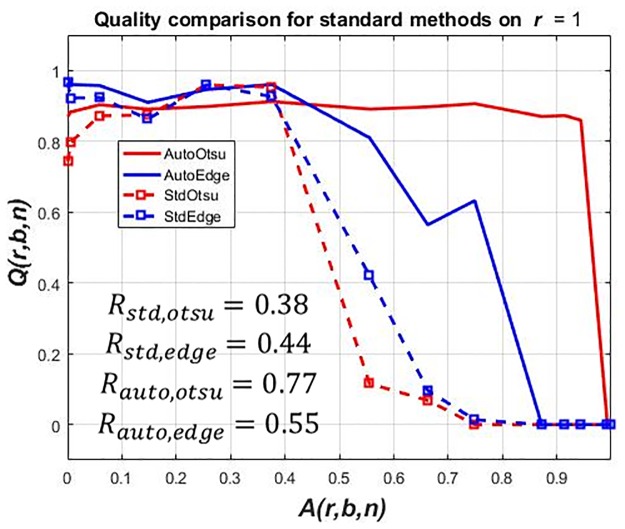
(Case 1) Results of image segmentation parameter adaptation (non-optimized) on benchmark data set *r* = 1. *Q*(*r*, *b*, *n*) vs. *A*(*r*, *b*, *n*). The non-optimized results from *r* = 1, 2, 3 are shown in S8, S9 and S10 Figs respectively. The overall effect of using the best result is not a glaring one. The difference is fairly small between optimized and non-optimized result using *δ* = 0.02 for automatic tuning of intensity threshold and *δ* = 0.01 for the edge detection threshold.

**Fig 9:**
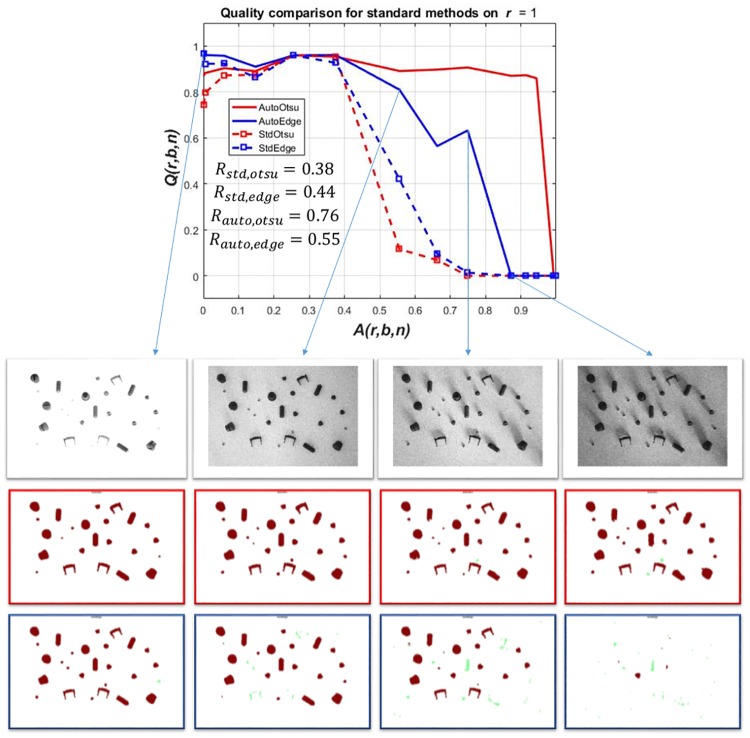
(Case 1) Results of image segmentation parameter adaptation (optimized) on benchmark data set *r* = 1. *Q*(*r*, *b*, *n*) vs. *A*(*r*, *b*, *n*). In first row, original images from data set *r* = 1 are given. The second row shows corresponding segmentation and classification results using parametric feedback tuning of Otsu segmentation (AutoOtsu). The third row shows corresponding segmentation and classification results using similar tuning of edge detection method (AutoEdge). Red and green colors represent correct and wrong classification of the segmented BLOB respectively w.r.t ground truth BLOB. The robustness values for each method are given in Table 1.

Moreover, even using a lower *δ* value does not completely ensure an improvement at each artifact level. The non-optimized version for *r* = 1 can be seen in Fig 8. The solution is to adapt the best value among both implementations. This is done by selecting the best result among standard feedforward and proposed feedback method at each artifact level of a scene. The optimized result can be seen in Fig 9.

Fig 9 shows a performance degradation in all methods indicated by *Q*(*r*, *b*, *n*) with increasing artifact levels *A*(*r*, *b*, *n*). To quantify the performance, the robustness measures *R*_std,otsu_, *R*_std,edge_, *R*_auto,otsu_ and *R*_auto,edge_ are given. *R*_std,otsu_ and *R*_std,edge_ are values of robustness *R* according to Eq (2) for StdOtsu and StdEdge respectively. *R*_auto,otsu_ and *R*_auto,edge_ are values of robustness *R* according to Eq (2) for AutoOtsu and AutoEdge respectively. Larger *R* values show higher robustness.

For the standard feedforward application *R*_std,otsu_ = 0.38 and *R*_std,edge_ = 0.44 were obtained. Using the automatic feedback parameter adaptation method, the performance of both methods can be improved especially at higher artifact levels. This is evident by robustness values of both methods i.e. *R*_auto,otsu_ = 0.76 and *R*_auto,edge_ = 0.55. Moreover, using parameter adaptation, the result at any artifact level for both methods cannot be worse than that of standard feedforward methods.

**Fig 10:**
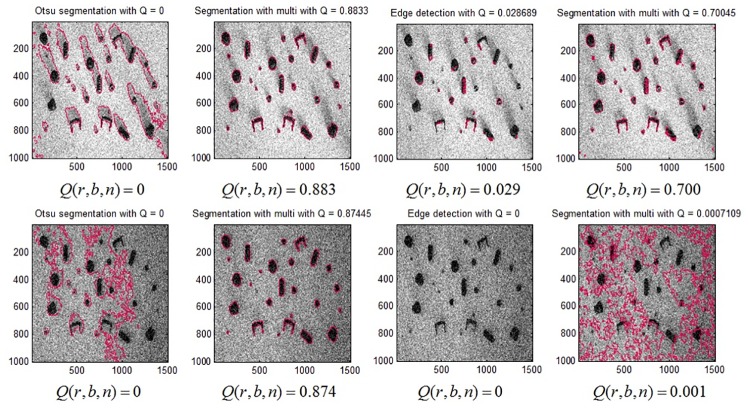
(Case 1) Segmentation outcome (shown in red) for Otsu segmentation and Sobel edge detection: feedforward (StdOtsu, StdEdge) and parameter adaptation (AutoOtsu, AutoEdge) at artifact level *A*(*r*, *b*, *n*), ≈ 0.75 in the first row and ≈ 0.94 in the second row. First column: segmentation result for StdOtsu. Second column: segmentation result for AutoOtsu. Third column: segmentation result for StdEdge. Fourth column: segmentation result for AutoEdge.

It is clear from the first two columns in Fig 10, that due to the presence of shadows erroneous BLOBs (that also delineate shadows) are found when the standard feedforward Otsu segmentation is used. Conversely, when the thresholding parameter is adapted, a better segmentation quality is obtained at high artifact levels. In the case of the Sobel edge detection, BLOBs found at the same artifact level are also compared using both techniques (i.e. feedforward vs. feedback parameter adaptation). Due to the presence of shadows, edges are not fully detected and a predefined image dilation parameter is not able to form all BLOBs resulting in a low *Q*(*r*, *b*, *n*) value using the standard feedforward application. Conversely, when *t*_edge_ is adapted iteratively using feedback, a better segmentation quality is obtained at high artifact level.

Wrong classification assignments occur often at extremely high artifact levels for edge detection due to incorrectly segmented objects. As an example, this effect can be observed in the images in first column (StdOtsu) and the last image (AutoEdge) of row 2 in Fig 10 where undesired object boundaries are obtained. Otsu segmentation is more efficient at higher artifact levels in comparison to Sobel edge detection (see first two images (StdOtsu, AutoOtsu) in comparison to last two images (StdEdge, AutoEdge) of row 2 in Fig 10).

The robustness of other scenes (i.e. *r* = 2, 3, 4) are given in S1, S2 and S3 Figs. Moreover, the mean and standard deviation of *Q*(*r*, *b*, *n*) for different scenes are given in S4 and S5 Figs respectively and for artifact levels in S6 and S7 Figs respectively.

To show the effect of tuning multiple parameters, only a single parameter for image intensity thresholding *t* was selected for manual tuning by an expert. However, for parameter adaptation, **p** = [*w* *t* *s*]^*T*^ was selected such that *w* = {3, 4, 5}, *s* = {3, 5, 7, 9, 11} and *t* was varied from *t*_*min*_ = 0.01 to *t*_*max*_ = 0.99 with *δ* = 0.04. The optimization was performed using *n*-dimensional grid search where *n* represents the number of parameters to be tuned and Eq (4) is obtained using all possible combinations of **p**. The features (*m* = 3) selected were: area *f*_*i*1_, eccentricity *f*_*i*2_ and solidity *f*_*i*3_. This was also done to show how much multiple parameter tuning can improve the results compared to single parameter tuning.

**Fig 11:**
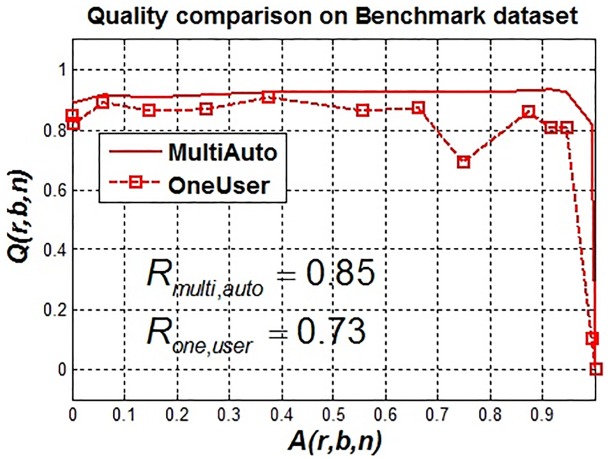
(Case 1) Segmentation outcome in terms of *Q*(*r*, *b*, *n*) against *A*(*r*, *b*, *n*): expert with one parameter (OneUser) vs. multiple parameter adaptation (MultiAuto). Dotted line shows expert segmentation outcome where as solid line shows multiple parameter adaptation. The robustness results are given in Table 1.

The tuning of single parameter by an expert was done manually using trial-and-error method and it took approximately half an hour to find the optimal parameter sets for each individual artifact level. However, this may not be possible using two additional parameters due to large number of possible parameter combinations and manually checking the outcome by trial-and-error method. The results of segmentation by a manual expert tuning one parameter versus automatic tuning of three different parameters simultaneously are given in Fig 11.

The expert segmentation based on *t* is quite good even with increasing noise but at very high artifact levels satisfactory results are not obtained. This is due to the fact that in circumstances of high artifact levels, more than one parameter may be required to be tuned by an expert in order to get satisfactory results. This may get very hectic without any guarantee of finding out the optimal parameter set. Therefore, one can automatically tune parameters to get a more robust outcome as shown in Fig 11.

### 3.2 Case 2

In many real applications, an absolute ground truth may not be available. Therefore, Case 2 is useful when a user wants to segment specific objects based on apriori abstract knowledge. This case can be applied to any kind of object as long as the object could be described by reasonable abstract features. In the benchmark data set, there are lot of different objects in a data set and a user wish to segment certain targeted object classes.

**Fig 12:**
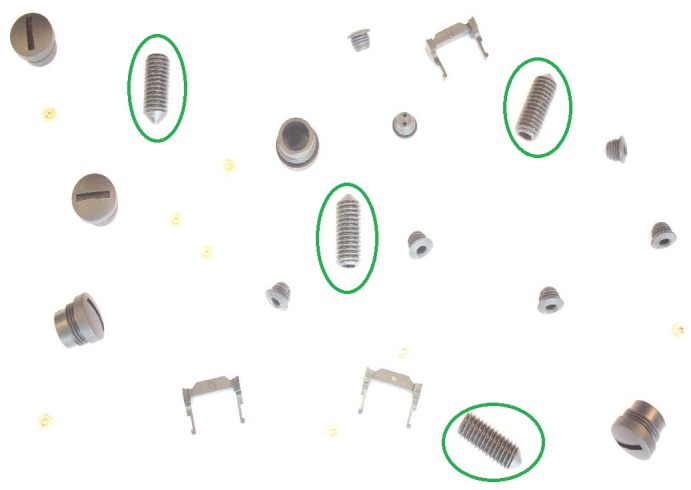
(Case 2) Object type to be found in the data set. Set screws encircled in green are to be found in *r* = 1.

**Fig 13:**
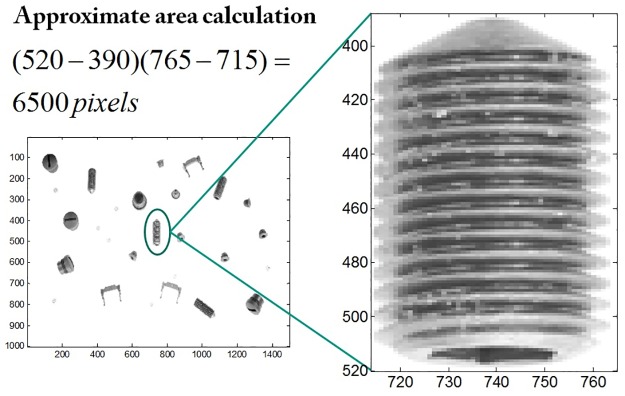
(Case 2) Estimating the area feature of a certain object. Set screws encircled in green on left and zoomed version on right to roughly calculate the number of pixels that constitute the area of a set screw.

This method also gives the possibility to define objects to be found, e.g. a user may want to find out a co-called “set screw” (encircled green in Fig 12) among all other objects in images of *r* = 1. However, it is intuitively clear to the user that this kind of objects are medium-sized, greater in eccentricity and have higher solidity. The features selected are area *f*_*i*1_ (in pixels), eccentricity *f*_*i*2_ and solidity *f*_*i*3_ such that *m* = 3. Using manually defined trapezoidal fuzzy membership functions, these features are represented using *θ* with (*a*_1_, *b*_1_, *c*_1_, *d*_1_) = (0, 5000, 7000, 8000), (*a*_2_, *b*_2_, *c*_2_, *d*_2_) = (0.6, 0.85, 0.95, 0.96) and (*a*_3_, *b*_3_, *c*_3_, *d*_3_) = (0.85, 0.95, 1, 1). The pixels describing the area of the set screws could easily be estimated by simply zooming at the screw object in noise free image as shown in the Fig 13. These features in our data set (*r* = 1) were derived from absolute ground truth and are among important features necessary to segment set screws. A user can use a noise-free image to extract the abstract features semi-automatically using the manual parametrization of image segmentation.

Moreover, *n*_*c*_ in *r* = 1 for set screws is 4. This can be formulated as well in our evaluation criterion using *θ*_*c*_(*n*_*c*_) with (*a*_4_, *b*_4_, *c*_4_, *d*_4_) = (2, 4, 4, 6). However, if *n*_*c*_ is known, *θ*_*c*_(*n*_*c*_) is equal to 1 and user can find only *n*_*c*_ objects that maximizes the criterion Eq (7) and subsequently evaluate **p**_opt,user_(*r*, *b*, *n*) according to Eq (4).

**p**_user_ = *t* was selected as shown in Fig 4. Firstly, **p**_opt,user_(*r*, *b*, *n*) gives us objects selected according to criterion Eq (7). Then, these objects are checked according to criterion Eq (4) to adopt an optimal parameter set. For Otsu’s method and Sobel edge detection, *t*_otsu_ and *t*_edge_ were adapted respectively. It is object-based since each detected object is compared against the reference features and the objects that maximize criterion Eq (7) are selected.

**Fig 14:**
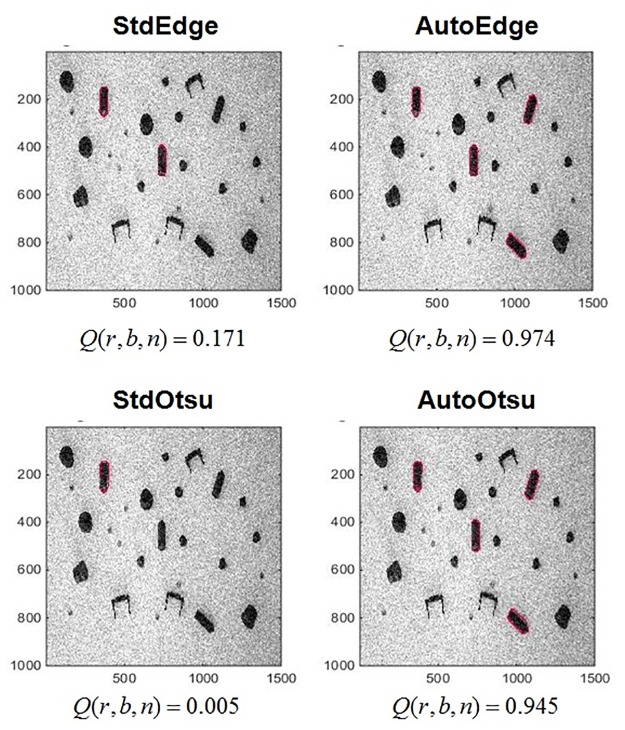
(Case 2) Segmentation outcome for Otsu segmentation and Sobel edge detection for abstract ground truth defined by end user using only one object type at *A*(*r*, *b*, *n*) = 0.5553. First and second columns represent feedforward and feedback application of both methods respectively. First and second rows show Sobel edge detection and Otsu segmentation results respectively. The red outlines show the boundaries of segmented objects.

The results for detecting set screws in *r* = 1 are first shown in terms of segmentation. This is done throughout the graded data set with increasing artifact levels. An example of image segmentation at a medium artifact level (*A*(*r*, *b*, *n*) = 0.55) is given in Fig 14. Otsu segmentation and Sobel edge detection are first applied in feedforward fashion and the results could be seen in the first column of Fig 14. Then, feedback adaptation of both methods is done and the improvement in results can be seen in the second column of Fig 14.

**Fig 15:**
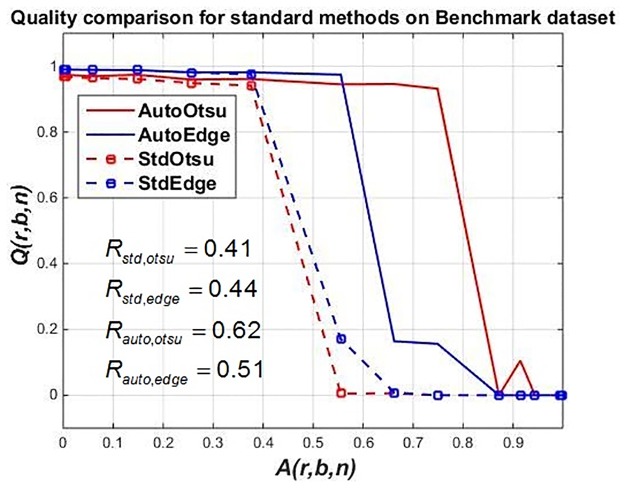
(Case 2) Segmentation outcome for Otsu segmentation and Sobel edge detection for abstract ground truth defined by end user using only one object type with increasing *A*(*r*, *b*, *n*). Dotted red and blue lines show outcome of Otsu segmentation and Sobel edge detection respectively with fixed parameter values respectively whereas solid red and blue lines show parameter adaptation using *t*. The robustness values for each method are given in Table 2.

This could be seen more intuitively by introducing later the classification scheme into the pipeline and the results (feedforward vs. feedback) are shown in Fig 15. It is clear from Fig 15 that as the artifact level is increased, it is hard to detect targeted objects based on features defined by user as an a priori knowledge. However, if we apply feedback parameter adaptation using **p** = *t* using both segmentation methods as described in Case 1, optimization is then done according to Eq (7).

The results for feedback are shown in solid lines for both the methods in Fig 15. It can be seen that there is a considerable improvement in results using parameter adaptation in the case of both segmentation methods when compared to feedforward application.

At *A*(*r*, *b*, *n*) ≥ 0.9, both methods perform poorly, no matter if we adapt parameters or not. This is due to the fact the optimization is clearly looking for certain objects with user-defined input reference features and with noise detected, feature extraction is greatly disturbed.

**Fig 16:**
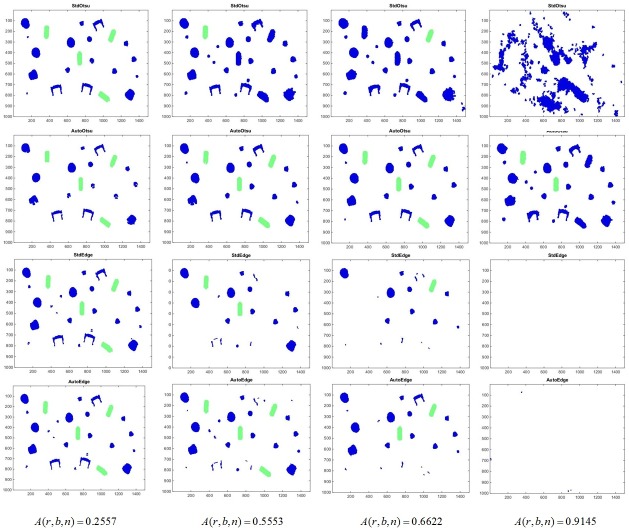
(Case 2) Segmentation/classification outcome for Otsu segmentation and Sobel edge detection of one object class using feedback method against increasing artifact levels along the columns from left to right. First row: StdOtsu. Second row: AutoOtsu. Third row: StdEdge. Fourth row: AutoEdge. Green color shows right classification of segments and blue color shows the objects that are not classified as set screws.

To show the effect of segmented objects on classification accuracy, Fig 16 is shown for different artifact levels in case of both segmentation methods with feedback parameter adaptation. It is evident in Fig 16, that as *A*(*r*, *b*, *n*) increases above 0.5, number of correctly classified objects gradually decreases using standard feedforward methods. However, robustness is increased by using feedback-based parameter adaptation (indicated by *R*_auto,otsu_ and *R*_auto,edge_ values in Table 2). At medium artifact levels, the segmentation results are seen to be improved when automatic parameter tuning is done (see second and third columns in Fig 16).

**Table 2:** Robustness values of standard segmentation methods (StdOtsu, StdEdge) vs. feedback adaptation with one parameter i.e. *t*_otsu_ for AutoOtsu and *t*_edge_ for AutoEdge (for abstract ground truth).

Algorithm type	Robustness symbol	Robustness value
StdOtsu	*R*_std,otsu_	0.41
StdEdge	*R*_std,edge_	0.44
AutoOtsu	*R*_auto,otsu_	0.62
AutoEdge	*R*_auto,edge_	0.51

## 4 Discussion

Our new method delivers two important application aspects: As shown in Case 1, the method can improve parameters based on an absolute ground truth. Therefore, a set of images can be marked manually by the user (e.g. in Photoshop) and optimal parameters can be derived without a detailed understanding of the image processing pipeline. Furthermore, the new parametrized routine is robust against noise and artifacts compared to common image processing routines.

Moreover, this case also helps an expert who is looking at the whole data set and trying to adapt optimal parameters for the images at varying artifact levels. Multiple parameter tuning done automatically can yield robust results for an expert in comparison to manual tuning of parameters which becomes quite hectic. Also, tuning multiple parameters automatically gives better results than tuning just a single parameter.

Case 2 introduces the possibility to describe an image set in a rather abstract fashion. A user may define targeted objects in terms of estimated a priori knowledge, e.g. estimated size and number of objects. Parameters are optimized to find these described properties.

Furthermore, in combination with a classifier, the method can also be used to extract a certain kind of object out of all segmented objects and therefore build an image processing pipeline being capable of picking object classes out of an image. Therefore, it is a new method for classification. This is a very efficient and automatic way to segment specific objects in a large data set and get them classified automatically.

The quality of the optimal image segmentation depends upon how well a certain targeted parameter is found. Non-linear optimization to find the targeted parameters should be used for better results instead of a exhaustive enumeration and grid search. As the number of parameters increase, the complexity increases and time taken to tune all parameters automatically grows exponentially. Therefore, grid search for bigger problems including large number of parameters is not recommended.

Additionally, feedback parameter adaptation can be further fine-tuned by making structural changes to image processing pipeline (such as increasing image pre-processing steps or removing it altogether etc.). The pipeline modifications would not affect the optimization process. However, one must choose a priori information carefully based on the the type of result expected from the chosen pipeline structure. Image normalization has not been done in this data set. Even without background noise removal, image segmentation quality has shown to be improved. Similarly, we induced only Gaussian noise and shading effects. The image normalization would, in this case, be less challenging as in case microscopic images containing inherent microscopic (both additive and multiplicative) noise. Therefore, with this data set, only parameter adaptation involved directly in image segmentation is adapted.

In addition to the induced artifacts, the performance and robustness of image processing routines would be investigated as a function of scene complexity in the near future. Moreover, application to hyperspectral and point cloud data would also be tested.

## Supporting Information

S1 Fig (Case 1) Results of image segmentation parameter adaptation (optimized) on benchmark data set *r* = 2. *Q*(*r*, *b*, *n*) vs. *A*(*r*, *b*, *n*). StdOtsu and StdEdge represent the standard feedforward implementation of Otsu thresholding and Sobel edge detection respectively. AutoOtsu and AutoEdge represent the automatic parameter adaptation of thresholding and Sobel edge detection method respectively.
(TIF)

S2 Fig (Case 1) Results of image segmentation parameter adaptation (optimized) on benchmark data set *r* = 3. *Q*(*r*, *b*, *n*) vs. *A*(*r*, *b*, *n*). StdOtsu and StdEdge represent the standard feedforward implementation of Otsu thresholding and Sobel edge detection respectively. AutoOtsu and AutoEdge represent the automatic parameter adaptation of thresholding and Sobel edge detection method respectively.
(TIF)

S3 Fig (Case 1) Results of image segmentation parameter adaptation (optimized) on benchmark data set *r* = 4. *Q*(*r*, *b*, *n*) vs. *A*(*r*, *b*, *n*). StdOtsu and StdEdge represent the standard feedforward implementation of Otsu thresholding and Sobel edge detection respectively. AutoOtsu and AutoEdge represent the automatic parameter adaptation of thresholding and Sobel edge detection method respectively.
(TIF)

S4 Fig Mean *μ* values of robustness *R* for all scenes *r*. (TIF)

S5 Fig Standard deviation *σ* in robustness *R* values for all scenes *r*. (TIF)

S6 Fig Mean *μ* values of quality *Q*(*r*, *b*, *n*) at each artifact level *A*(*r*, *b*, *n*). (TIF)

S7 Fig Standard deviation *σ* in quality *Q*(*r*, *b*, *n*) values at each artifact level *A*(*r*, *b*, *n*). (TIF)

S8 Fig (Case 1) Results of image segmentation parameter adaptation (non-optimized) on benchmark data set *r* = 2. *Q*(*r*, *b*, *n*) vs. *A*(*r*, *b*, *n*). StdOtsu and StdEdge represent the standard feedforward implementation of Otsu thresholding and Sobel edge detection respectively. AutoOtsu and AutoEdge represent the automatic parameter adaptation of thresholding and Sobel edge detection method respectively.
(TIF)

S9 Fig (Case 1) Results of image segmentation parameter adaptation (non-optimized) on benchmark data set *r* = 3. *Q*(*r*, *b*, *n*) vs. *A*(*r*, *b*, *n*). StdOtsu and StdEdge represent the standard feedforward implementation of Otsu thresholding and Sobel edge detection respectively. AutoOtsu and AutoEdge represent the automatic parameter adaptation of thresholding and Sobel edge detection method respectively.
(TIF)

S10 Fig (Case 1) Results of image segmentation parameter adaptation (non-optimized) on benchmark data set *r* = 4. *Q*(*r*, *b*, *n*) vs. *A*(*r*, *b*, *n*). StdOtsu and StdEdge represent the standard feedforward implementation of Otsu thresholding and Sobel edge detection respectively. AutoOtsu and AutoEdge represent the automatic parameter adaptation of thresholding and Sobel edge detection method respectively.
(TIF)
