# Supplementary figures and images for: A New Feedback-Based Method for Parameter Adaptation in Image Processing Routines
Source: PLoS One. 2016 Oct 20;11(10):e0165180. doi: 10.1371/journal.pone.0165180 (PMC5072585; doi:10.1371/journal.pone.0165180)

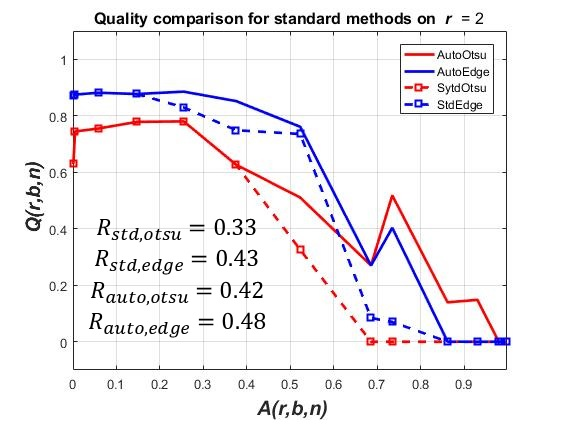

Supplement: S1 Fig — Q(r, b, n) vs. A(r, b, n). StdOtsu and StdEdge represent the standard feedforward implementation of Otsu thresholding and Sobel edge detection respectively. AutoOtsu and AutoEdge represent the automatic parameter adaptation of thresholding and Sobel edge detection method respectively. (TIF) [file pone.0165180.s001.tif]

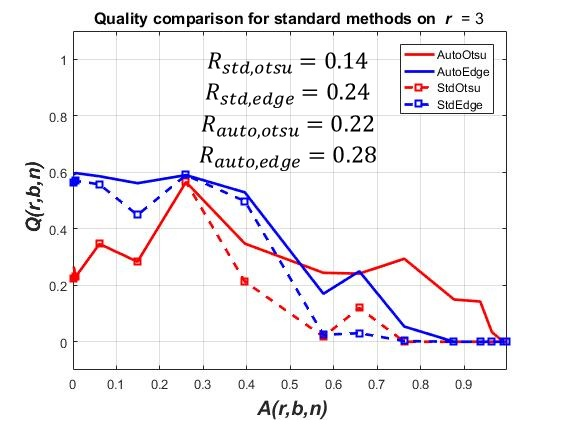

Supplement: S2 Fig — Q(r, b, n) vs. A(r, b, n). StdOtsu and StdEdge represent the standard feedforward implementation of Otsu thresholding and Sobel edge detection respectively. AutoOtsu and AutoEdge represent the automatic parameter adaptation of thresholding and Sobel edge detection method respectively. (TIF) [file pone.0165180.s002.tif]

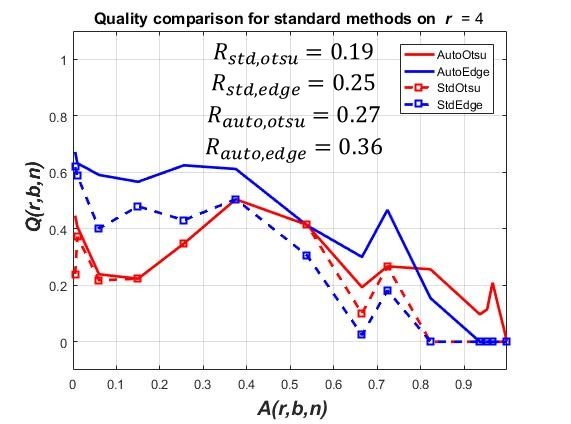

Supplement: S3 Fig — Q(r, b, n) vs. A(r, b, n). StdOtsu and StdEdge represent the standard feedforward implementation of Otsu thresholding and Sobel edge detection respectively. AutoOtsu and AutoEdge represent the automatic parameter adaptation of thresholding and Sobel edge detection method respectively. (TIF) [file pone.0165180.s003.tif]

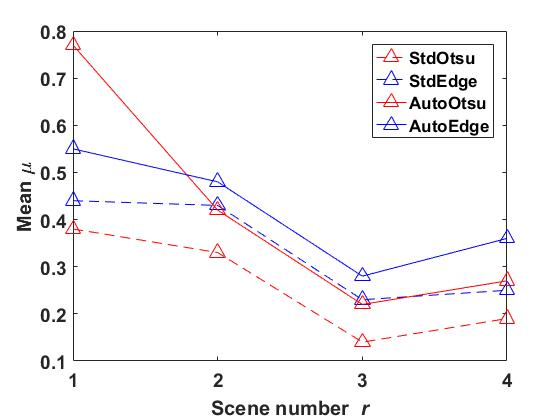

Supplement: S4 Fig — (TIF) [file pone.0165180.s004.tif]

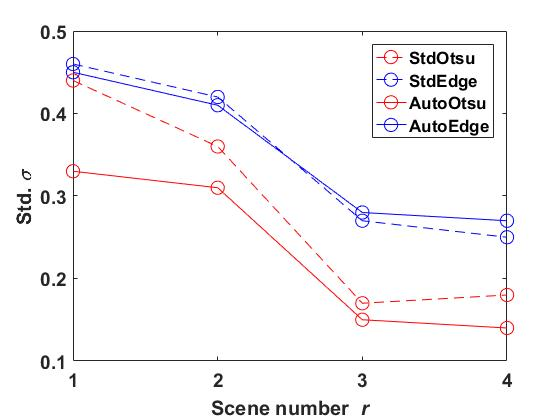

Supplement: S5 Fig — (TIF) [file pone.0165180.s005.tif]

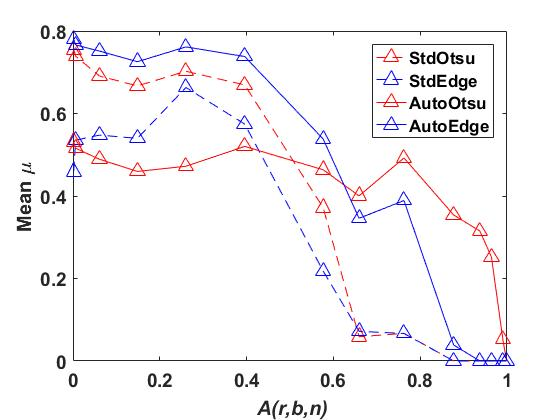

Supplement: S6 Fig — (TIF) [file pone.0165180.s006.tif]

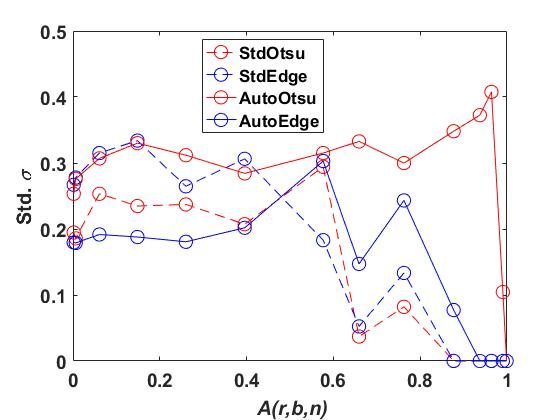

Supplement: S7 Fig — (TIF) [file pone.0165180.s007.tif]

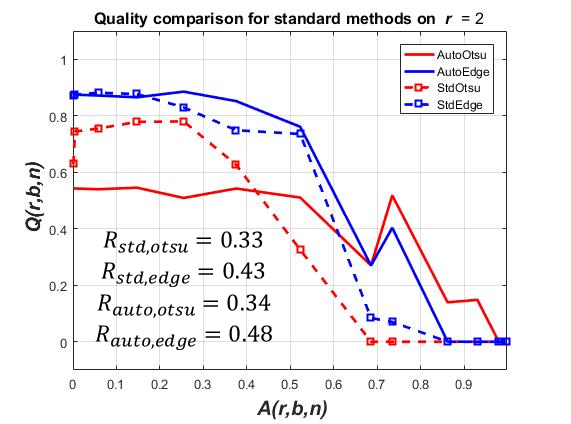

Supplement: S8 Fig — Q(r, b, n) vs. A(r, b, n). StdOtsu and StdEdge represent the standard feedforward implementation of Otsu thresholding and Sobel edge detection respectively. AutoOtsu and AutoEdge represent the automatic parameter adaptation of thresholding and Sobel edge detection method respectively. (TIF) [file pone.0165180.s008.tif]

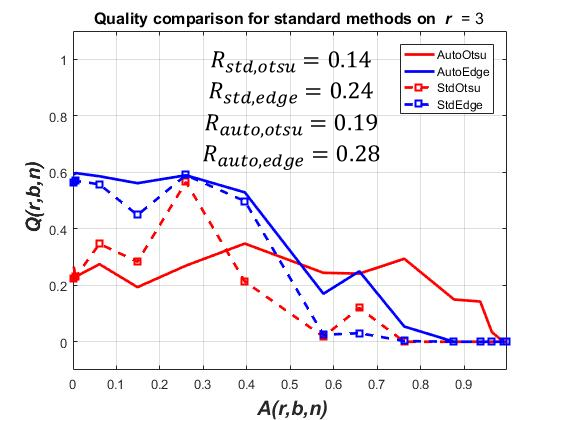

Supplement: S9 Fig — Q(r, b, n) vs. A(r, b, n). StdOtsu and StdEdge represent the standard feedforward implementation of Otsu thresholding and Sobel edge detection respectively. AutoOtsu and AutoEdge represent the automatic parameter adaptation of thresholding and Sobel edge detection method respectively. (TIF) [file pone.0165180.s009.tif]

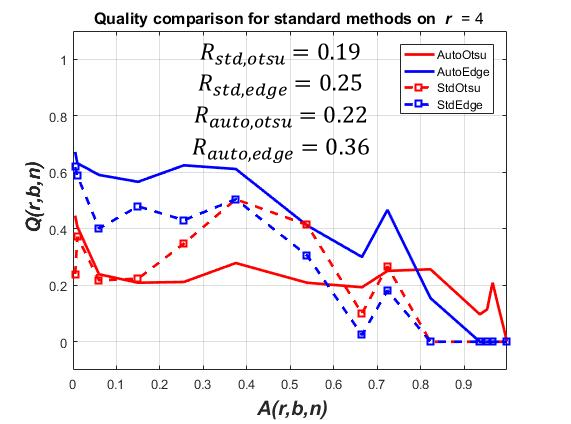

Supplement: S10 Fig — Q(r, b, n) vs. A(r, b, n). StdOtsu and StdEdge represent the standard feedforward implementation of Otsu thresholding and Sobel edge detection respectively. AutoOtsu and AutoEdge represent the automatic parameter adaptation of thresholding and Sobel edge detection method respectively. (TIF) [file pone.0165180.s010.tif]
